# Association between an improved positioning management strategy and non-incisional low back pain and sleep quality in patients undergoing modified radical mastectomy for breast cancer: a retrospective cohort study

**DOI:** 10.3389/fpain.2026.1831922

**Published:** 2026-10-27

**Authors:** Huajing Sun, Mei Zhang, Shanshan Feng, Wenhai Zhang, Zihao Liu, Chunfeng Liang, Yukun Liu, Xiaohong Sun

**Affiliations:** 1 Department of Breast Surgery, Qingdao Central Hospital, University of Health and Rehabilitation Sciences, Qingdao, Shandong, China; 2 Department of Breast Surgery, Eighth Affiliated Hospital of Guangxi Medical University, Guigang City People’s Hospital, Guigang, Guangxi, China; 3 Department of Breast Surgery, Guangxi Medical University Tumor Hospital, Nanning, Guangxi, China; 4 Department of Gynecology, Qingdao Central Hospital, University of Health and Rehabilitation Sciences, Qingdao, Shandong, China

**Keywords:** breast cancer surgery, low back pain, non-incisional low back pain, positioning management, sleep quality

## Abstract

**Background:**

Non-incisional low back pain, particularly low back pain, and sleep disturbance are common yet underrecognized problems during early recovery after breast cancer surgery. This study evaluated whether an improved positioning management strategy was associated with reduced non-incisional low back pain and improved postoperative sleep quality.

**Methods:**

This single-center retrospective cohort study included adult women who underwent modified radical mastectomy or simple mastectomy for breast cancer between January 2024 and December 2025. Patients were grouped according to whether an improved positioning management strategy was implemented during the perioperative period (intervention vs. control). Non-incisional low back pain was assessed using the Numerical Rating Scale (NRS, 0–10), and sleep quality was assessed using the Richards–Campbell Sleep Questionnaire (RCSQ: 0–100) at 24, 48, and 72 h postoperatively. Safety outcomes included total drainage volume during the first 3 postoperative days, subcutaneous seroma, and skin-flap ischemia or necrosis. Linear mixed-effects models with random intercepts were used for repeated measures, adjusting for age, body mass index (BMI), analgesic regimen, and postoperative nausea and vomiting (PONV), with Holm correction for multiple comparisons. Gamma regression and Firth-penalized logistic regression were applied for safety outcomes.

**Results:**

Baseline characteristics were generally comparable between groups, although BMI was slightly higher in the intervention group (*P* = .04) and was adjusted for in multivariable analyses. Improved positioning management was associated with significantly lower non-incisional low back pain (*β* = −0.711, *P* < .001). Pain increased over time in the control group, whereas significant group-by-time interactions indicated greater pain reduction in the intervention group at 48 h (*β* = −0.358, *P* = .002) and 72 h (*β* = −0.737, *P* < .001). Between-group differences in pain at all time points remained significant after Holm correction. The association remained significant after additional adjustment for incisional pain. Improved positioning management was also associated with higher postoperative sleep quality, with significant group and group-by-time effects at 24–72 h. No significant between-group differences were observed in drainage volume, seroma, or flap ischemia/necrosis.

**Conclusion:**

An improved positioning management strategy was associated with reduced early postoperative non-incisional low back pain and better sleep quality after breast cancer surgery, without evidence of increased drainage- or flap-related complications. These findings should be interpreted as observational associations and warrant confirmation in prospective studies.

## Background

Breast cancer is among the most common malignancies in women worldwide (1, 2), and surgical treatment still plays a central role within multidisciplinary management strategies (3, 4). With continued advances in surgical techniques, anesthetic management, and the concept of enhanced recovery after surgery (ERAS) (5–7), perioperative mortality and major complications in patients undergoing breast cancer surgery have declined substantially (8, 9). Nevertheless, postoperative pain, discomfort, and delayed functional recovery remain important barriers to early quality of life and the overall recovery experience (10–12).

Perioperative rehabilitation within enhanced recovery after surgery (ERAS) pathways is increasingly recognized as an important component of postoperative recovery after breast cancer surgery. Core ERAS interventions generally include preoperative optimization, patient education, standardized multimodal analgesia, early oral intake, early ambulation, and structured functional recovery. In this context, early mobilization and structured postoperative functional recovery are central ERAS elements that may reduce postoperative complications, promote independence, and improve the recovery experience (13). Therefore, a focused review of ERAS-based rehabilitation should emphasize perioperative optimization, early mobilization, and functional recovery as the core background for improving postoperative recovery after breast cancer surgery.

Previous research on postoperative pain after breast cancer surgery has primarily focused on the assessment and management of incisional pain, such as regional blocks (10, 14–17), patient-controlled analgesia (18, 19), and multimodal analgesic strategies (20, 21). However, in clinical practice, a considerable proportion of patients also experience early postoperative pain that has no direct anatomic association with the surgical incision, with non-incisional low back pain being particularly common (22, 23). For the purpose of this study, non-incisional low back pain refers to pain that is not anatomically associated with the surgical incision itself. This type of pain is commonly related to prolonged intraoperative and postoperative positioning, which may induce postural muscular strain, sustained soft-tissue compression, microcirculatory impairment, and peripheral nociceptive sensitization. In patients undergoing breast cancer surgery, restricted mobility, drainage tubes, and fear of movement-related pain may further aggravate non-incisional low back pain during the early postoperative period. This type of pain is often related to intraoperative positioning, restricted postoperative mobility, and prolonged maintenance of the supine position, yet it has received insufficient attention in previous studies.

Existing evidence suggests that prolonged fixed positioning may cause sustained soft-tissue compression, abnormal muscle tension, and microcirculatory impairment, thereby triggering or exacerbating truncal pain (24, 25). For patients after breast cancer surgery, operative-site restrictions, the presence of drainage tubes, and fear of pain may further extend the duration of positional limitation, making non-incisional low back pain an important factor affecting comfort and early mobilization. However, systematic evaluation and interventional research targeting non-incisional low back pain after breast cancer surgery remain limited.

In addition to pain, postoperative sleep deterioration is also a common but often overlooked problem among patients with breast cancer (26). Previous studies have shown that postoperative pain, discomfort, nausea and vomiting, and psychological stress can disrupt sleep architecture (27), while sleep disturbance may further delay postoperative recovery by increasing pain sensitivity and impairing emotional regulation and immune function (28, 29). Therefore, from a comprehensive rehabilitation perspective, it is clinically meaningful to address both pain control and sleep quality improvement.

In recent years, nonpharmacological nursing interventions have received increasing attention as adjunctive strategies for improving postoperative comfort and recovery. Among these interventions, positioning management represents a practical bedside strategy that may influence not only patient comfort but also local pressure distribution, drainage-tube stability, and protection of the operative area (30). In patients undergoing breast cancer surgery, postoperative positioning is clinically important because the operative side often contains drainage tubes, a vulnerable skin flap, and axillary or chest-wall wound areas that require protection from excessive compression or traction. However, although targeted positioning may reduce sustained lumbosacral pressure and improve comfort, inappropriate or overly aggressive repositioning could theoretically disturb wound drainage, increase fluid accumulation, or compromise flap perfusion. Therefore, evaluating positioning management after breast cancer surgery requires simultaneous assessment of symptom-related outcomes and safety outcomes.

From a safety perspective, the selection of drainage- and flap-related outcomes is clinically relevant to targeted positioning interventions. After breast cancer surgery, drainage tubes are commonly placed to evacuate postoperative fluid and reduce dead-space accumulation. Repositioning maneuvers or support-pad placement, if not carefully performed, may theoretically kink, compress, or pull drainage tubes, thereby affecting drainage function and increasing the risk of fluid accumulation or subcutaneous seroma. Similarly, excessive pressure on the operative-side chest wall, axillary region, or skin flap may impair local microcirculation and flap perfusion, potentially contributing to skin-flap ischemia or necrosis. Therefore, total drainage volume, postoperative subcutaneous seroma, and skin-flap ischemia or necrosis were selected as clinically relevant safety outcomes to determine whether improved positioning management could be implemented without compromising surgical-site stability.

Moreover, in routine clinical practice, improved positioning management is often implemented pragmatically rather than through strict randomization; therefore, its effectiveness and safety warrant verification in retrospective or cohort studies. Clarifying the impact of this strategy on pain, sleep, and safety outcomes within the context of standard perioperative care is important for optimizing postoperative nursing pathways and improving the quality of recovery after breast cancer surgery.

Accordingly, this retrospective cohort study was designed with three core objectives. The primary objective was to evaluate whether an improved positioning management strategy was associated with reduced early postoperative non-incisional low back pain after breast cancer surgery, as measured by Numerical Rating Scale (NRS) scores at 24, 48, and 72 h postoperatively. The secondary objective was to assess whether this strategy was associated with improved postoperative sleep quality, as measured by Richards–Campbell Sleep Questionnaire (RCSQ) scores over the same early postoperative period. The safety objective was to determine whether the strategy was associated with increased drainage- or flap-related complications, as assessed by total drainage volume during the first 3 postoperative days, postoperative subcutaneous seroma, and skin-flap ischemia or necrosis. By linking each outcome measure to a prespecified clinical objective, this study aimed to provide clinically interpretable evidence for optimizing postoperative nursing care after breast cancer surgery.

## Materials and methods

### Study design and participants

This single-center retrospective cohort study included consecutive eligible female patients who underwent modified radical mastectomy (MRM) or simple mastectomy (SM) for breast cancer at a tertiary hospital between January 2024 and December 2025.

Patients were assigned to the control or intervention group according to the period during which they underwent surgery and whether the improved positioning management strategy had been implemented as part of the standardized perioperative nursing protocol. Specifically, patients treated before the standardized implementation of the improved positioning management strategy received routine postoperative positioning care and were classified as the control group, whereas patients treated after implementation received the improved positioning management strategy and were classified as the intervention group. The allocation was therefore based on a chronological change in departmental nursing practice rather than on clinician preference or individual patient characteristics. Apart from positioning management, both groups received the same routine perioperative treatment and nursing care. The study protocol was approved by the Medical Ethics Committee of Guangxi Medical University Cancer Hospital (Approval No. KY2026002). Given the retrospective nature of the study and the use of previously collected de-identified clinical data, the requirement for written informed consent was waived by the ethics committee.

### Inclusion and exclusion criteria

Inclusion criteria were as follows: (1) female patients aged ≥18 years; (2) pathologically confirmed breast cancer; (3) receipt of MRM or SM; (4) complete postoperative records of non-incisional low back pain scores and sleep quality assessments; and (5) completion of at least one follow-up assessment at 24, 48, or 72 h postoperatively.

Exclusion criteria were: (1) a documented history of significant chronic low back pain or spinal disorders before surgery; (2) long-term use of analgesics or sedative–hypnotics before surgery; (3) severe psychiatric disorders or cognitive impairment precluding completion of subjective rating scales; and (4) incomplete medical or nursing records, including missing key baseline variables, missing primary or secondary outcome assessments, or unavailable safety outcome records.

### Sample size and power consideration

Because this was a retrospective cohort study, the sample size was determined by the number of eligible patients who underwent breast cancer surgery during the predefined study period from January 2024 to December 2025. All patients who met the inclusion and exclusion criteria were included to minimize selection bias. A sample size adequacy assessment was subsequently performed based on the primary outcome, namely non-incisional low back pain measured by the NRS.

Assuming a two-sided significance level of *α* = 0.05, 80% statistical power, a 1:1 allocation ratio between the control and intervention groups, and a small-to-moderate standardized effect size of Cohen's *d* = 0.30 for the between-group difference in postoperative non-incisional low back pain, at least 176 patients per group were required. The final cohort included 240 patients in the control group and 240 patients in the intervention group, exceeding the minimum required sample size. Under the same assumptions, the available sample size provided approximately 90% power to detect a between-group difference corresponding to Cohen's *d* = 0.30. Therefore, the final cohort size was considered sufficient for evaluating the primary outcome.

### Positioning management strategies

Control group. Patients received routine postoperative positioning care, primarily in the supine position, with nonsystematic adjustments based on patient-reported comfort; no structured or continuous positioning intervention was implemented.

Intervention group. In addition to routine care, patients in the intervention group received an improved positioning management strategy delivered by uniformly trained nursing staff. After returning to the ward, patients were initially placed in a supine position with the head of the bed elevated by approximately 15°–30°, according to vital signs, recovery from anesthesia, and patient comfort. Soft pillows, wedge cushions, or foam positioning pads were used to support the lower back, knees, and shoulder–back region, with the aim of reducing sustained lumbosacral pressure and postural muscular strain. Once vital signs were stable, small-range repositioning maneuvers were performed approximately every 2 h, provided that the drainage tubes were not pulled and that compression of the operative-side skin flap and axillary region was avoided. These maneuvers included mild left or right lateral tilting of approximately 15°–30° and readjustment of support pads. For patients who could not tolerate obvious turning, micro-positioning adjustments were used, such as changing pelvic tilt, modifying knee-support height, or shifting the lower-back support point. All positioning procedures were individualized according to patient-reported pain, comfort, drainage-tube location, and protection of the operative side.

### Data collection and quality control

Clinical data were extracted retrospectively from the electronic medical record system, anesthesia records, surgical records, and routine nursing documentation. Before data extraction, a standardized data abstraction form was developed to ensure consistency across reviewers. The extracted variables were predefined and included demographic characteristics, surgical procedure, axillary dissection, anesthesia technique, postoperative analgesic regimen, postoperative nausea and vomiting, non-incisional low back pain scores, sleep quality scores, drainage volume, subcutaneous seroma, and skin-flap ischemia or necrosis.

Two trained reviewers independently extracted the data using the standardized abstraction form. Before formal data extraction, the reviewers were trained on variable definitions, outcome definitions, and data entry rules. Extracted data were cross-checked between reviewers, and any discrepancies were resolved by rechecking the original medical or nursing records. If disagreement remained, a senior investigator adjudicated the final value. To ensure data accuracy, implausible values and outliers, such as unusually high drainage volume or inconsistent postoperative assessment times, were verified against the original source records.

Missing data were handled according to predefined rules. Patients with missing primary outcome data, missing key sleep quality assessments, unavailable safety outcome records, or incomplete baseline covariates required for multivariable analysis were excluded from the final analysis. No imputation was performed for the primary or secondary outcomes because all patients included in the final cohort had complete NRS and RCSQ assessments at 24, 48, and 72 h postoperatively. All data were de-identified before analysis to protect patient confidentiality.

### Outcome measures

Primary outcome. The primary clinical objective was to evaluate the association between improved positioning management and early postoperative non-incisional low back pain. Therefore, non-incisional low back pain was selected as the primary outcome and was assessed using the Numerical Rating Scale (NRS; 0–10) at 24, 48, and 72 h postoperatively; higher scores indicated more severe pain. The NRS was chosen because it provides a simple and clinically interpretable measure of patient-reported pain intensity, and the 24–72-h assessment window was selected to capture the early postoperative period during which positioning-related discomfort is most likely to occur.

Secondary outcome. The secondary clinical objective was to determine whether improved positioning management was associated with better early postoperative sleep quality. Sleep quality was therefore selected as the secondary outcome and was assessed using the Richards–Campbell Sleep Questionnaire (RCSQ; 0–100) at 24, 48, and 72 h postoperatively; higher scores indicated better sleep quality. The RCSQ was used because it captures patient-perceived sleep depth, latency, awakenings, return to sleep, and overall sleep quality, which are clinically relevant dimensions of early postoperative recovery.

Safety outcomes. The safety objective was to evaluate whether improved positioning management could be implemented without increasing drainage- or flap-related complications. Accordingly, safety endpoints included: (1) total drainage volume during the first 3 postoperative days (mL), which reflected early postoperative drainage burden and potential disturbance of wound drainage; (2) occurrence of postoperative subcutaneous seroma, which was used to assess fluid accumulation potentially related to inadequate drainage or local tissue disruption; and (3) occurrence of skin-flap ischemia or necrosis, which was selected to assess whether repositioning or support-pad placement might compromise local flap perfusion. These outcomes were chosen because positioning interventions involve repeated support adjustment and small-range repositioning, which may theoretically affect drainage-tube stability, local pressure distribution, and flap blood supply.

NRS and RCSQ assessments were performed according to routine ward nursing procedures at predefined postoperative time points and were recorded in standardized nursing documentation. Safety outcomes were obtained from postoperative nursing records, drainage charts, surgical records, and physician progress notes. Because the intervention involved a nursing improved positioning management strategy, blinding of the healthcare providers delivering and recording the intervention was not feasible in routine clinical practice. However, standardized assessment instruments, predefined assessment time points, standardized data abstraction, and source-record verification were used to reduce variability and improve data reliability.

### Covariates

Collected covariates included age and body mass index (BMI). Surgery-related variables comprised surgical procedure (MRM vs. SM) and whether axillary dissection was performed. Anesthesia/analgesia-related variables included anesthesia technique (general anesthesia alone vs. general anesthesia combined with thoracic paravertebral block) and postoperative analgesic regimen (Protocol A vs. Protocol B, categorized by analgesic combinations). Perioperative complications included postoperative nausea and vomiting (PONV) within 24 h after surgery. Incisional pain scores were additionally collected for sensitivity analyses.

Potential confounders were considered at both the design and analysis stages. At the design stage, patients with a documented history of significant chronic low back pain, spinal disorders, long-term analgesic use, or long-term sedative–hypnotic use before surgery were excluded to reduce confounding from pre-existing pain or medication-related factors. Perioperative variables collected included type of surgery, axillary lymph node dissection, anesthesia technique, postoperative analgesic regimen, and PONV within 24 h after surgery. These variables were compared between groups at baseline. Age, BMI, analgesic regimen, and PONV were included in the main multivariable models according to clinical relevance and model stability, while type of surgery and axillary lymph node dissection were additionally included in the models for safety outcomes. Duration of surgery was considered a potential confounder; however, because this variable was incompletely documented in the retrospective records, it was not included in the primary models.

### Link between study objectives and outcome measures

The outcome measures were selected to correspond directly to the prespecified study objectives. The NRS score for non-incisional low back pain was used to quantify the primary symptom targeted by the positioning intervention. The RCSQ score was used to quantify sleep quality, which was considered a clinically relevant recovery-related outcome potentially influenced by pain relief and improved comfort. Drainage volume, subcutaneous seroma, and skin-flap ischemia or necrosis were selected as safety outcomes because the intervention involved repeated repositioning and support-pad adjustment, which could theoretically influence drainage function, local tissue pressure, and flap perfusion.

### Statistical analysis

Continuous variables are presented as mean ± standard deviation (SD) or median [interquartile range (IQR)] depending on distribution. Between-group comparisons were performed using the Student *t* test or the Mann–Whitney *U* test, as appropriate. Categorical variables are presented as counts (percentages) and were compared using the *χ*^2^ test or Fisher exact test.

Linear mixed-effects models (LMMs) were used to evaluate trajectories of non-incisional low back pain and sleep quality over time. A random intercept for each patient (1|id) was included. Fixed effects comprised group, time, and the group-by-time interaction, with adjustment for covariates including age, BMI, analgesic regimen, and PONV. Degrees of freedom were estimated using the Satterthwaite or Kenward–Roger method. Marginal mean differences were derived from the fitted models, with Holm correction applied for multiple comparisons.

Total drainage volume over the first 3 postoperative days was analyzed using gamma regression with a log link. For low-incidence events (subcutaneous seroma and skin-flap ischemia/necrosis), Firth's penalized logistic regression was applied to reduce small-sample bias. All analyses were two-sided, and *P* < 0.05 was considered statistically significant. Statistical analyses were conducted using R software (version 4.5.2).

To mitigate potential selection bias and confounding inherent to the retrospective before-after cohort design, baseline demographic, surgical, anesthesia-related, and postoperative clinical characteristics were compared between groups. Variables considered clinically relevant or potentially associated with the outcomes, including age, BMI, analgesic regimen, and PONV, were included as covariates in the multivariable models.

The sample size adequacy assessment was performed using a two-sample comparison framework for the primary outcome, with a two-sided significance level of α = 0.05 and 80% power.

Analyses were performed on the final complete-case cohort after exclusion of patients with missing key baseline, outcome, or safety data. Because the final cohort had complete repeated assessments for the primary and secondary outcomes, no outcome imputation was performed.

## Results

### Baseline characteristics

#### Follow-up completeness

A total of 480 eligible patients were included in the final analysis, including 240 patients in the control group and 240 patients in the intervention group. All included patients completed postoperative outcome assessments at 24, 48, and 72 h. Therefore, no attrition occurred during the predefined follow-up period. Linear mixed-effects models were nevertheless applied to appropriately account for repeated-measure correlations across time points.

As shown in Table 1, there were no statistically significant differences between the intervention and control groups with respect to age, preoperative self-reported sleep status, type of surgery, performance of axillary dissection, anesthesia technique, postoperative analgesic regimen, postoperative nausea and vomiting (PONV) within 24 h, or total drainage volume during the first 3 postoperative days (all *P* > 0.05), indicating overall comparability between groups. Body mass index (BMI) differed modestly between groups, with a slightly higher BMI in the intervention group (*P* = 0.038); therefore, BMI was included as a covariate in subsequent multivariable models.

**Table 1:** Baseline characteristics of the study population.

Characteristic	Group		*P* value
	Control (*n* = 240)	Intervention (*n* = 240)	
Age, years, mean ± SD	52 ± 10	52 ± 10	0.635
Body mass index (BMI), kg/m^2^, mean ± SD	24.0 ± 3.4	24.6 ± 3.2	0.038
Baseline sleep score (0–10), median (Q1, Q3)	6.25 (5.00, 7.40)	6.30 (4.55, 7.60)	0.801
Total drainage volume within 3 days, mL, median (Q1, Q3)	373 (266, 488)	365 (276, 468)	0.757
Type of surgery, *n* (%)			0.739
Modified radical mastectomy (MRM)	187 (77.9)	190 (79.2)	
Simple mastectomy (SM)	53 (22.1)	50 (20.8)	
Axillary lymph node dissection, *n* (%)			0.75
No	57 (23.8)	60 (25.0)	
Yes	183 (76.3)	180 (75.0)	
Anesthesia method, *n* (%)			0.268
General anesthesia (GA)	176 (73.3)	165 (68.8)	
GA + paravertebral block (PVB)	64 (26.7)	75 (31.3)	
Postoperative analgesia regimen, *n* (%)			0.142
PCIA regimen A	139 (57.9)	123 (51.3)	
PCIA regimen B	101 (42.1)	117 (48.8)	
Postoperative nausea and vomiting within 24 h, *n* (%)			0.375
No	192 (80.0)	184 (76.7)	
Yes	48 (20.0)	56 (23.3)	
Seroma, *n* (%)			0.873
No	219 (91.3)	218 (90.8)	
Yes	21 (8.8)	22 (9.2)	
Flap necrosis, *n* (%)			0.19
No	226 (94.2)	232 (96.7)	
Yes	14 (5.8)	8 (3.3)	

PCIA, patient-controlled intravenous analgesia; PVB, paravertebral block; PONV, postoperative nausea and vomiting.

### Linear mixed-effects model results for non-incisional low back pain

Table 2 presents the linear mixed-effects model (LMM) results for non-incisional low back pain without adjustment for incisional pain. After adjustment for age, BMI, and postoperative analgesic regimen, the intervention group had significantly lower non-incisional low back pain scores than the control group (*β* = −0.711, *P* < 0.001), indicating that the improved positioning management strategy was associated with reduced postoperative non-incisional low back pain.

**Table 2:** Linear mixed-effects model for non-incisional low back pain (NRS) after surgery (unadjusted for incisional pain).

Variable	*β* estimate	SE	df	*t* value	*P* value
Intercept	0.351	0.333	498.3	1.054	0.292
Intervention group (vs. control group)	−0.711	0.099	1,150	−7.149	<0.001
Time 48 h (vs. 24 h)	0.408	0.081	915	5.04	<0.001
Time 72 h (vs. 24 h)	0.51	0.08	910.9	6.347	<0.001
Age (per 1-year increase)	0.0288	0.0036	476.1	7.919	<0.001
Body mass index (per 1 kg/m^2^ increase)	0.1045	0.0112	481.6	9.357	<0.001
Analgesia regimen (PCIA-B vs. PCIA-A)	−0.117	0.0739	479.3	−1.583	0.114
Intervention × time (48 h)	−0.358	0.115	920.7	−3.099	0.002
Intervention × time (72 h)	−0.737	0.115	920.1	−6.391	<0.001

*β* coefficients represent fixed-effect estimates from the linear mixed-effects model. A negative *β* value indicates a reduction in non-incisional low back pain intensity.

The model included a random intercept for each patient (1 | subject ID), and degrees of freedom were estimated using the Satterthwaite approximation.

NRS, numerical rating scale; PCIA, patient-controlled intravenous analgesia.

Time also had a significant effect on pain scores. Compared with 24 h postoperatively, non-incisional low back pain scores were significantly higher at 48 and 72 h (both *P* < 0.001), suggesting a worsening trend over time under routine care.

Moreover, a significant group-by-time interaction was observed. Compared with the control group, the intervention group showed significantly lower pain scores at 48 and 72 h (intervention × time at 48 h: *β* = −0.358, *P* = 0.002; intervention × time at 72 h: *β* = −0.737, *P* < 0.001), with a more pronounced interaction at 72 h. Model-based marginal means are shown in Figure 1.

**Figure 1:**
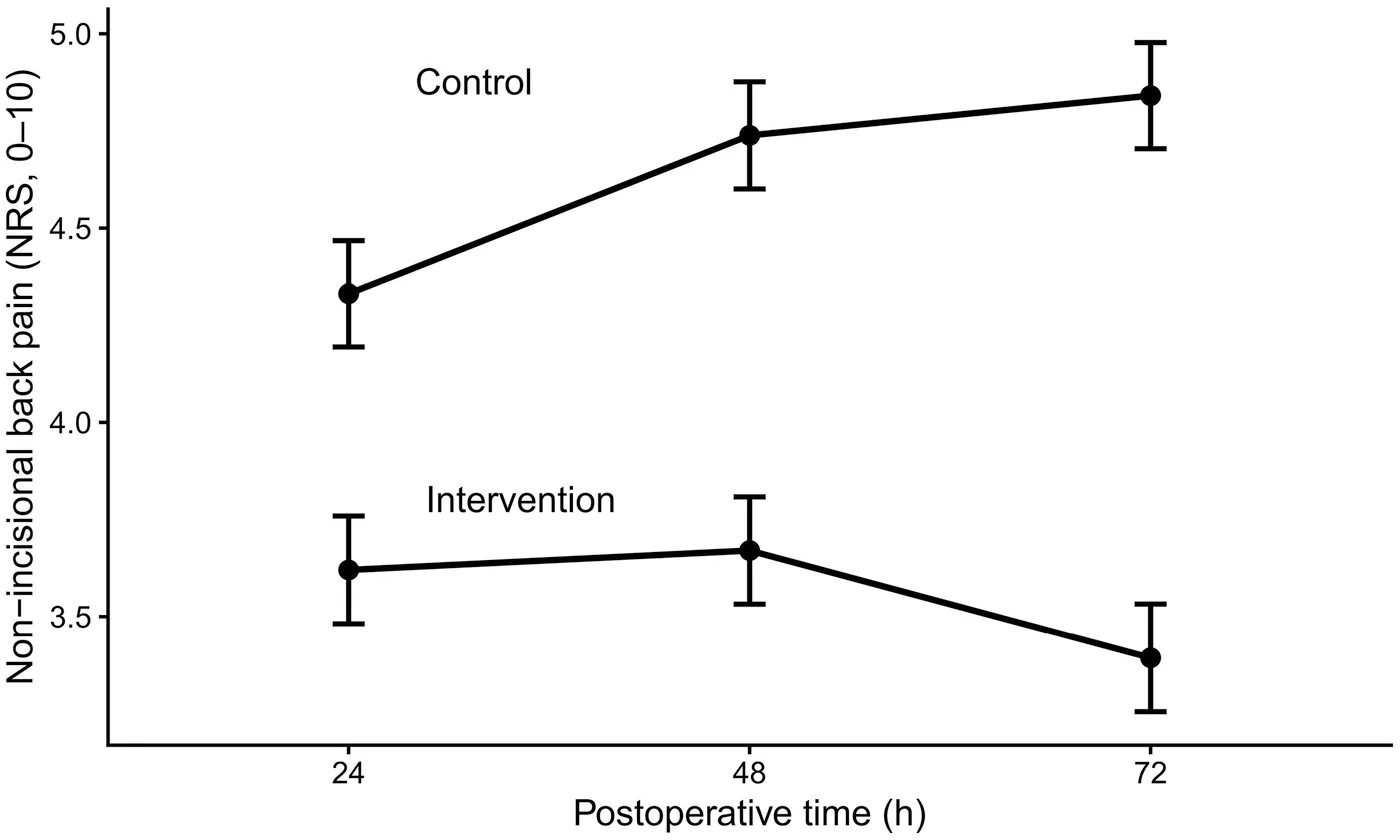
Trajectory of non-incisional low back pain over time. Changes in non-incisional low back pain scores (numerical rating scale, NRS; 0–10) at 24, 48, and 72 h after surgery in the control and intervention groups. Data are presented as mean values with error bars indicating standard errors.

As illustrated in Figure 1, the control group exhibited a gradual increase in non-incisional low back pain from 24 to 72 h, whereas the intervention group maintained consistently lower pain scores, with the between-group difference widening over time. Specifically, pain scores in the intervention group were lower than those in the control group at 24, 48, and 72 h, supporting the effectiveness of the improved positioning management strategy in alleviating early postoperative non-incisional low back pain after breast cancer surgery.

Age and BMI were important covariates associated with pain severity. Each 1-year increase in age was associated with higher pain scores (*β* = 0.0288, *P* < 0.001), and each 1 kg/m^2^ increase in BMI was also associated with higher pain scores (*β* = 0.1045, *P* < 0.001). The difference between analgesic regimens (PCIA_B vs. PCIA_A) was not statistically significant (*P* = 0.114).

### Between-group comparisons of non-incisional low back pain at each time point

Model-based marginal mean comparisons are shown in Table 3. At 24, 48, and 72 h postoperatively, the intervention group had significantly lower non-incisional low back pain scores than the control group, and the differences remained significant after Holm adjustment (all adjusted *P* < 0.001).

**Table 3:** Between-group differences in non-incisional low back pain (NRS) at each postoperative time point (marginal mean comparison, intervention minus control).

Time point	Estimated difference (points)	SE	df	*t* value	Adjusted P value^†^	Direction of effect
24 h	−0.711	0.0994	1,146	−7.149	<0.001	Lower in intervention group
48 h	−1.068	0.0995	1,148	−10.733	<0.001	Lower in intervention group
72 h	−1.447	0.0994	1,145	−14.566	<0.001	Lower in intervention group

Data are presented as marginal mean differences derived from the linear mixed-effects model. Negative values indicate lower non-incisional low back pain scores in the intervention group compared with the control group.

Degrees of freedom were estimated using the Kenward–Roger approximation. Results were averaged across levels of postoperative analgesia regimen.

NRS, numerical rating scale.

† *P* values were adjusted for multiple comparisons using the Holm method.

Notably, the reduction in pain was evident as early as 24 h and became larger at 48 and 72 h, suggesting that the benefit of improved positioning management emerged early and strengthened over time. The estimated marginal mean differences were negative at all three time points, consistent with the progressively diverging pain trajectories shown in Figure 1.

From a clinical perspective, the overall adjusted group effect for non-incisional low back pain (*β* = −0.711) was smaller than the more conservative MCID threshold of approximately 2 points on the 0–10 NRS. However, previous studies of musculoskeletal pain have also suggested that a reduction of approximately 1 point, or about 15% from baseline, may represent a minimal clinically important improvement. In the present study, the model-based between-group differences increased from −0.711 points at 24 h to −1.068 points at 48 h and −1.447 points at 72 h, indicating that the later postoperative differences reached or exceeded the lower range of reported MCID values.

### Sensitivity analysis: LMM adjusted for incisional pain

To determine whether the association between improved positioning management and non-incisional low back pain was independent of incisional pain, the original model was further adjusted for incisional pain NRS scores; results are presented in Table 4.

**Table 4:** Linear mixed-effects model for non-incisional low back pain (NRS) adjusted for incisional pain.

Variable	*β* estimate	SE	df	*t* value	*P* value
Intercept	0.561	0.38	737.1	1.478	0.14
Intervention group (vs. control group)	−0.709	0.099	1,150	−7.132	<0.001
Time 48 h (vs. 24 h)	0.391	0.082	924.1	4.749	<0.001
Time 72 h (vs. 24 h)	0.476	0.086	943.4	5.56	<0.001
Incisional pain (NRS)	−0.033	0.0287	1,196	−1.147	0.252
Age (per 1-year increase)	0.0289	0.0036	475.8	7.931	<0.001
Body mass index (per 1 kg/m^2^ increase)	0.1042	0.0112	481.6	9.341	<0.001
Analgesia regimen (PCIA-B vs. PCIA-A)	−0.119	0.0738	479.3	−1.605	0.109
Intervention × time (48 h)	−0.361	0.116	919.9	−3.124	0.002
Intervention × time (72 h)	−0.737	0.115	919.2	−6.389	<0.001

*β* coefficients represent fixed-effect estimates from the linear mixed-effects model. Negative *β* values indicate lower non-incisional low back pain intensity.

The model included a random intercept for each participant (1 | subject ID). Degrees of freedom were estimated using the Satterthwaite approximation.

NRS, numerical rating scale; PCIA, patient-controlled intravenous analgesia.

After adjustment for incisional pain, the intervention group continued to demonstrate significantly lower non-incisional low back pain scores than the control group, and the main effect of group remained statistically significant, indicating that the benefit of improved positioning management was not explained by differences in incisional pain severity. The time effect also remained significant: compared with 24 h, non-incisional low back pain scores were higher at 48 and 72 h, suggesting a worsening trend over time under routine care. Importantly, the association between incisional pain NRS scores and non-incisional low back pain did not reach statistical significance, implying that incisional pain was not the primary driver of changes in non-incisional low back pain.

The group-by-time interaction remained statistically significant after adjustment for incisional pain, with significantly lower pain scores in the intervention group at 48 and 72 h and a stronger effect at 72 h. Age and BMI remained significant covariates, with higher age and BMI associated with increased pain scores. Differences between analgesic regimens were not statistically significant.

### Effect of improved positioning management on postoperative sleep quality

The LMM results evaluating sleep quality are shown in Table 5. After adjustment for age, BMI, PONV within 24 h, and analgesic regimen, the intervention group had significantly higher sleep quality scores than the control group, indicating that improved positioning management was associated with better early postoperative sleep quality.

**Table 5:** Linear mixed-effects model for sleep quality (RCSQ).

Variable	*β* estimate	SE	df	*t* value	*P* value
Intercept	37.65	3.77	485.6	9.982	<0.001
Intervention group (vs. control group)	7.9	1.06	1,011	7.492	<0.001
Time 48 h (vs. 24 h)	4.18	0.79	904.5	5.321	<0.001
Time 72 h (vs. 24 h)	7.57	0.78	901.2	9.706	<0.001
Age (per 1-year increase)	−0.098	0.041	468.7	−2.378	0.018
Body mass index (per 1 kg/m^2^ increase)	−0.193	0.126	473.4	−1.530	0.127
Postoperative nausea and vomiting within 24 h (yes vs. no)	−4.67	1.02	476.4	−4.604	<0.001
Analgesia regimen (PCIA-B vs. PCIA-A)	1.26	0.83	471.2	1.51	0.132
Intervention × time (48 h)	3.28	1.12	909.3	2.926	0.003
Intervention × time (72 h)	3.12	1.12	908.9	2.784	0.005

*β* coefficients represent fixed-effect estimates from the linear mixed-effects model. Positive *β* values indicate improved sleep quality.

The model included a random intercept for each participant (1 | subject ID). Degrees of freedom were estimated using the Satterthwaite approximation.

RCSQ, Richards–Campbell Sleep Questionnaire; PCIA, patient-controlled intravenous analgesia; PONV, postoperative nausea and vomiting.

Time had a significant effect on sleep quality: compared with 24 h, sleep quality scores improved at 48 and 72 h, suggesting gradual recovery of sleep quality during the early postoperative period. A significant group-by-time interaction was also observed. Compared with the control group, the intervention group showed higher sleep quality scores at 48 and 72 h, with a more apparent between-group difference after 48 h, consistent with the trajectories shown in Figure 2.

**Figure 2:**
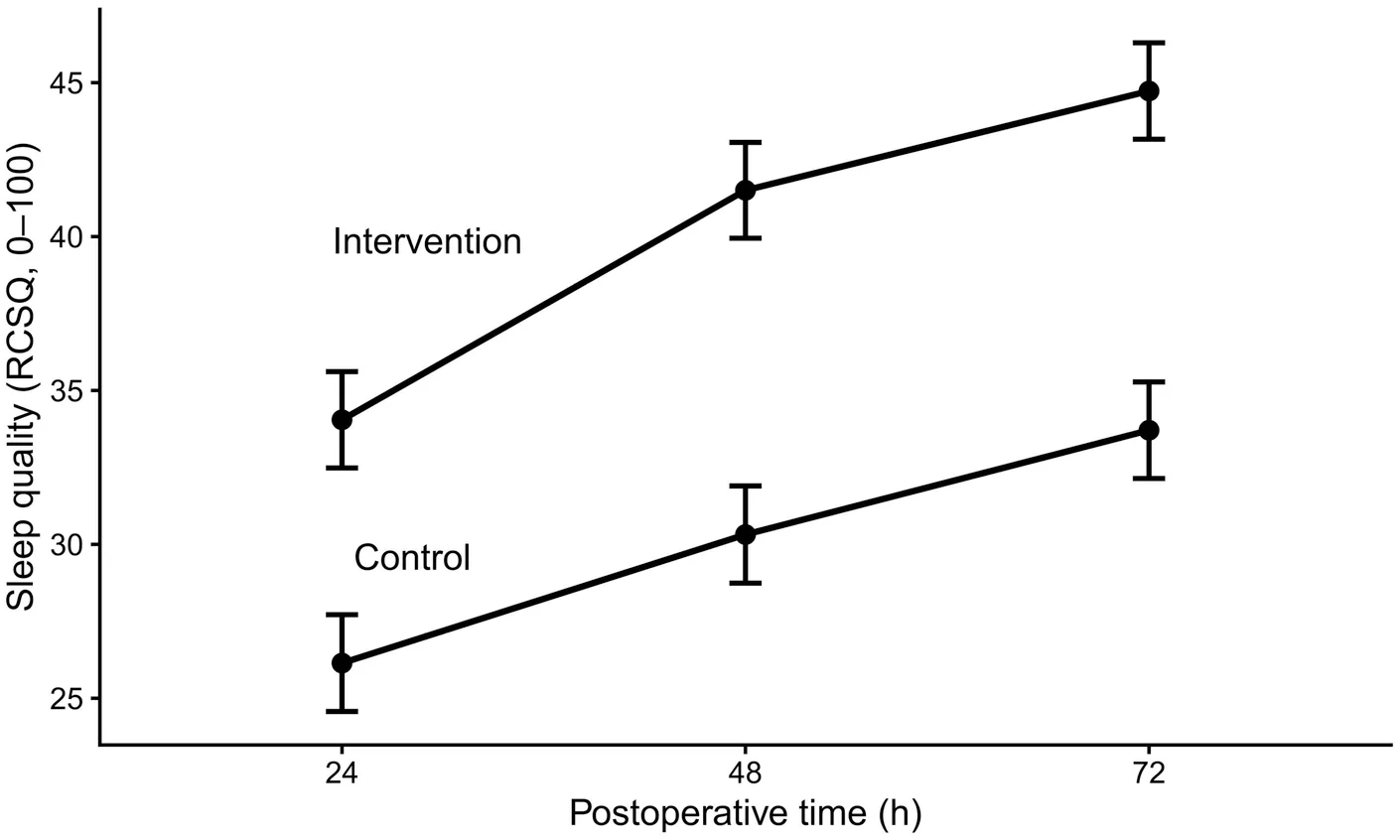
Trajectory of postoperative sleep quality over time. Changes in sleep quality assessed by the Richards–Campbell Sleep Questionnaire (RCSQ; 0–100) at 24, 48, and 72 h after surgery in the control and intervention groups. Data are presented as mean values with error bars indicating standard errors.

Covariate analyses indicated that age was negatively associated with sleep quality, suggesting poorer postoperative sleep among older patients. The occurrence of PONV within 24 h was associated with significantly lower sleep quality, highlighting PONV as an important adverse factor. BMI and analgesic regimen were not significantly associated with sleep quality.

### Between-group comparisons of sleep quality at each time point

Model-based marginal mean comparisons are presented in Table 6. At 24, 48, and 72 h, sleep quality scores were significantly higher in the intervention group than in the control group, and the differences remained statistically significant after Holm correction.

**Table 6:** Between-group differences in sleep quality (RCSQ) at each postoperative time point (marginal mean comparison, intervention minus control).

Time point	Estimated difference (points)	SE	df	*t* value	Adjusted *P* value^†^	Direction of effect
24 h	7.9	1.06	1,013	7.492	<0.001	Higher in intervention group
48 h	11.2	1.06	1,015	10.587	<0.001	Higher in intervention group
72 h	11	1.05	1,012	10.45	<0.001	Higher in intervention group

Data are presented as marginal mean differences derived from the linear mixed-effects model. Positive values indicate higher sleep quality scores in the intervention group compared with the control group.

Degrees of freedom were estimated using the Kenward–Roger approximation.

RCSQ, Richards–Campbell Sleep Questionnaire.

† *P* values were adjusted for multiple comparisons using the Holm method.

Specifically, the intervention group demonstrated better sleep quality as early as 24 h, with the between-group difference increasing at 48 and 72 h, suggesting an early and sustained beneficial association of improved positioning management with postoperative sleep quality. The estimated marginal mean differences were positive at all time points, consistent with the diverging trajectories shown in Figure 2.

### Total drainage volume during the first 3 postoperative days

Gamma regression (log link) results are shown in Table 7. After adjustment for age, BMI, type of surgery, and axillary dissection, there was no statistically significant difference between groups in total drainage volume during the first 3 postoperative days, suggesting that improved positioning management did not increase the risk of higher drainage output.

**Table 7:** Safety outcome 1: multivariable gamma regression analysis for total drainage volume within 3 postoperative days (log link).

Variable	*β* (log scale)	SE	*z* value	*P* value
Intervention group (vs. control group)	−0.036	0.0355	−1.02	0.309
Age (per 1-year increase)	−0.0006	0.0017	−0.35	0.727
Body mass index (per 1 kg/m^2^ increase)	0.0415	0.00533	7.78	<0.001
Type of surgery (simple mastectomy vs. modified radical mastectomy)	−0.070	0.0431	−1.63	0.104
Axillary lymph node dissection (yes vs. no)	0.0655	0.0412	1.59	0.112

*β* coefficients are presented on the logarithmic scale from a Gamma regression model with a log link function.

Positive *β* values indicate higher total drainage volume within 3 postoperative days.

The multivariable model was adjusted for age, body mass index, type of surgery, and axillary lymph node dissection.

BMI was positively associated with total drainage volume, indicating that patients with higher BMI were more likely to have increased drainage output. Age, type of surgery, and axillary dissection were not significantly associated with drainage volume.

### Risk of postoperative subcutaneous seroma

Firth's penalized logistic regression results are shown in Table 8. There was no statistically significant difference between groups in the risk of postoperative subcutaneous seroma, suggesting no increased seroma risk associated with improved positioning management.

**Table 8:** Safety outcome 2: multivariable firth penalized logistic regression analysis for seroma formation.

Variable	Odds ratio (OR)	95% CI	*P* value
Intervention group (vs. control group)	0.953	0.505–1.800	0.882
Age (per 1-year increase)	1.014	0.982–1.046	0.399
Body mass index (per 1 kg/m^2^ increase)	1.185	1.076–1.309	<0.001
Type of surgery (simple mastectomy vs. modified radical mastectomy)	1.322	0.619–2.655	0.456
Axillary lymph node dissection (yes vs. no)	1.865	0.834–4.854	0.135

Odds ratios were estimated using a multivariable Firth penalized logistic regression model to reduce small-sample bias.

An odds ratio >1 indicates an increased risk of seroma formation.

The model was adjusted for age, body mass index, type of surgery, and axillary lymph node dissection.

BMI was positively associated with seroma risk: each 1 kg/m^2^ increase in BMI was associated with a higher risk of seroma. Age, type of surgery, and axillary dissection were not significantly associated with seroma risk.

### Risk of skin-flap ischemia/necrosis

Firth's penalized logistic regression results are shown in Table 9. There was no statistically significant difference between groups in the risk of skin-flap ischemia or necrosis, suggesting that improved positioning management did not increase flap-related complications.

**Table 9:** Safety outcome 3: multivariable firth penalized logistic regression analysis for flap ischemia/necrosis.

Variable	Odds ratio (OR)	95% CI	*P* value
Intervention group (vs. control group)	0.571	0.230–1.337	0.199
Age (per 1-year increase)	0.973	0.932–1.016	0.217
Body mass index (per 1 kg/m^2^ increase)	1.014	0.894–1.148	0.832
Type of surgery (simple mastectomy vs. modified radical mastectomy)	0.25	0.028–0.990	0.048
Axillary lymph node dissection (yes vs. no)	1.071	0.423–3.164	0.892

Odds ratios were estimated using a multivariable Firth penalized logistic regression model to reduce small-sample bias.

An odds ratio <1 indicates a lower risk of flap ischemia or necrosis.

The model was adjusted for age, body mass index, type of surgery, and axillary lymph node dissection.

Type of surgery was significantly associated with flap ischemia/necrosis risk: compared with modified radical mastectomy, simple mastectomy was associated with a lower risk of flap ischemia/necrosis. Age, BMI, and axillary dissection were not significantly associated with flap ischemia/necrosis risk.

## Discussion

In this retrospective cohort study of patients undergoing breast cancer surgery, the improved positioning management strategy was associated with lower early postoperative non-incisional low back pain and better sleep quality during the first 72 postoperative hours. These two findings directly address the main clinical focus of the study: whether a structured, bedside positioning strategy can improve patient-reported comfort and sleep-related recovery after surgery. The model-based between-group differences in non-incisional low back pain increased from −0.711 points at 24 h to −1.068 points at 48 h and −1.447 points at 72 h, indicating that the benefit of positioning management became more evident as postoperative immobility and sustained supine positioning continued. Importantly, these symptom-related benefits were observed without an increase in drainage- or flap-related complications, supporting the clinical feasibility of this strategy when implemented with careful protection of drainage tubes and the operative side. Because of the retrospective observational design, these findings should be interpreted as associations rather than causal effects.

The reduction in non-incisional low back pain is clinically plausible because the intervention directly targeted posture-related sources of discomfort. Patients after breast cancer surgery often remain in a supine or semi-recumbent position because of wound protection, drainage tubes, fear of movement, and limited early mobility (31, 32). Prolonged fixed positioning may increase lumbosacral pressure and paraspinal muscle tension, thereby contributing to non-incisional low back pain (33). In the intervention group, scheduled small-range repositioning and individualized support of the lower back, knees, and shoulder–back region may have reduced sustained pressure and muscle strain (34). The widening between-group difference from 24 to 72 h further supports the interpretation that repeated positioning support may have cumulative benefits during the early postoperative period. Clinically, this finding suggests that non-incisional low back pain should be assessed separately from incisional pain and addressed through targeted nursing strategies rather than relying solely on pharmacologic analgesia.

The improvement in sleep quality should also be interpreted in relation to the positioning intervention and the pain findings (35). Sleep during the first postoperative days is commonly disrupted by pain, restricted mobility, drainage tubes, unfamiliar ward routines, and discomfort from prolonged supine positioning (36). By reducing non-incisional low back pain and improving body support, the intervention may have helped patients maintain a more comfortable resting posture and reduced nocturnal awakenings related to positional discomfort. The parallel improvement in pain and RCSQ scores suggests that postoperative sleep quality may be partly responsive to nonpharmacological comfort-oriented nursing care. For clinical practice, this means that sleep recovery after breast cancer surgery should not be viewed only as a pharmacologic or psychological issue; it may also be improved by systematic attention to posture, support surfaces, and safe repositioning (34).

Compared with previous positioning-related studies, the present study has a more specific symptom-oriented focus. Earlier studies of postoperative positioning have commonly emphasized pressure injury prevention, comfort nursing, pulmonary function, or early mobilization (34, 37). These outcomes are important, but they do not directly address non-incisional low back pain after breast cancer surgery. Our study extends this literature by focusing on a common but underrecognized symptom that is anatomically separate from the surgical incision and by simultaneously evaluating sleep quality as a patient-centered recovery outcome. In addition, by including drainage volume, subcutaneous seroma, and skin-flap ischemia or necrosis, the study addresses an important safety concern: whether repeated small-range repositioning can be performed without compromising surgical-site stability (34, 38). Thus, compared with previous positioning studies that mainly evaluated general comfort or complication prevention, our study provides more targeted evidence on symptom relief and sleep-related recovery in the specific context of breast cancer surgery.

Safety is a prerequisite for the adoption of any nursing intervention. Our data help address concerns that frequent positional changes might increase drainage output or compromise flap healing. Both gamma regression and logistic regression analyses indicated no increase in total drainage volume or flap-related complications associated with improved positioning management. These findings suggest that micro-positioning under standardized training does not disrupt surgical-site stability. Importantly, covariate analyses identified BMI as a factor associated with both non-incisional low back pain and safety outcomes, which is consistent with basic biomechanical considerations: patients with higher BMI may experience greater pressure gradients on the back in the supine position, increasing the risk of microcirculatory compromise (39). Therefore, in patients with obesity, improved positioning management may represent not only comfort-focused care but also a potentially important preventive approach against pressure-related tissue injury and ischemic pain.

The clinical implication of these findings is that improved positioning management may be incorporated into routine postoperative nursing pathways as a structured, low-cost, nonpharmacological strategy. Key elements include early identification of non-incisional low back pain, individualized support of the lumbosacral and shoulder–back regions, small-range repositioning, avoidance of operative-side compression, and continuous protection of drainage tubes. Rather than replacing analgesic treatment, this strategy may complement multimodal postoperative care by addressing posture-related discomfort that is not fully relieved by incision-focused analgesia.

Several limitations should be acknowledged. First, due to the retrospective observational nature of the study, residual confounding and selection bias cannot be fully excluded despite multivariable adjustment. Because the grouping was based on a chronological change in departmental nursing practice rather than random allocation, potential time-related bias and confounding from unmeasured changes in perioperative care may also exist. Second, although several clinically relevant covariates were collected and adjusted for, some perioperative variables, such as duration of surgery, were incompletely documented in the retrospective records and could not be included in the primary models. Therefore, residual confounding from unmeasured or incompletely recorded perioperative factors cannot be fully excluded. Third, the specific implementation of positioning management may have varied across individuals and may not be fully quantifiable. Moreover, because the intervention was a nursing-based improved positioning management strategy, blinding of healthcare providers responsible for outcome assessment was not feasible. As the primary and secondary outcomes were based on patient-reported subjective scales, the possibility of measurement bias cannot be completely excluded. Fourth, although the improved positioning management strategy does not require complex equipment, it requires standardized staff training, repeated repositioning maneuvers, and ongoing monitoring, which may increase nursing workload and indirect implementation costs. In addition, although standardized data abstraction and cross-checking procedures were used, formal inter-rater reliability coefficients were not calculated for all extracted variables, which may be considered a limitation of the retrospective data collection process. Furthermore, non-incisional low back pain and sleep quality were assessed only during the first 72 postoperative hours; therefore, whether the observed benefits persist beyond the early recovery period remains unknown. Finally, this was a single-center study with a relatively concentrated patient population, and the findings may not be fully representative of other hospitals, nursing-resource settings, or the broader population of postoperative breast cancer patients. Future multicenter and prospective studies are warranted to validate these findings and to further evaluate the scalability, resource requirements, and cost-effectiveness of this positioning management strategy.

## Conclusion

In conclusion, in this retrospective cohort study, implementation of an improved positioning management strategy was associated with lower early postoperative non-incisional low back pain and better sleep quality in patients undergoing breast cancer surgery, without an observed increase in postoperative drainage- or flap-related complications. These findings support further prospective evaluation of positioning management as a potential component of comprehensive perioperative nursing care.

## Data Availability

The original contributions presented in the study are included in the article/Supplementary Material, further inquiries can be directed to the corresponding author.

## References

[1] Giaquinto AN Sung H Newman LA Freedman RA Smith RA Star J. Breast cancer statistics 2024. CA Cancer J Clin. (2024) 74(6):477–95. 10.3322/caac.21863 39352042

[2] Kim J Harper A McCormack V Sung H Houssami N Morgan E. Global patterns and trends in breast cancer incidence and mortality across 185 countries. Nat Med. (2025) 31:1154–62. 10.1038/s41591-025-03502-3 39994475

[3] Anderson BO. Breast conservation therapy (BCT): surgery as the cornerstone of multi-specialty care. Breast Cancer. (2000) 7(4):273–5. 10.1007/BF02966388 11114848

[4] Eskandar K. The evolving role of surgery in multimodal cancer treatment: a comprehensive review. Surg Oncol. (2024) 14(4):55–62. 10.17650/2949-5857-2024-14-4-55-62

[5] Temple-Oberle C Shea-Budgell MA Tan M Semple JL Schrag C Barreto M. Consensus review of optimal perioperative care in breast reconstruction: enhanced recovery after surgery (ERAS) society recommendations. Plast Reconstr Surg. (2017) 139(5):1056e–71e. 10.1097/PRS.0000000000003242 28445352 PMC9074892

[6] Offodile AC Gu C Boukovalas S Coroneos CJ Chatterjee A Largo RD. Enhanced recovery after surgery (ERAS) pathways in breast reconstruction: systematic review and meta-analysis of the literature. Breast Cancer Res Treat. (2019) 173(1):65–77. 10.1007/s10549-018-4991-8 30306426

[7] Bian HZ Liau MYQ Cheong GPC Goo JTT Hwee JJ Chia CLK. Enhanced recovery after surgery for breast reconstruction—a systematic review and meta-analysis. Ann Breast Surg. (2024) 8:26. 10.21037/abs-23-44

[8] El-Tamer MB Ward BM Schifftner T Neumayer L Khuri S Henderson W. Morbidity and mortality following breast cancer surgery in women: national benchmarks for standards of care. Ann Surg. (2007) 245(5):665–71. 10.1097/01.sla.0000245833.48399.9a 17457156 PMC1877061

[9] Chatzidaki P Mellos C Briese V Mylonas I. Perioperative complications of breast cancer surgery in elderly women (≥80 years). Ann Surg Oncol. (2011) 18(4):923–31. 10.1245/s10434-010-1389-1 21107743

[10] Chiu M Bryson GL Lui A Watters JM Taljaard M Nathan HJ. Reducing persistent postoperative pain and disability 1 year after breast cancer surgery: a randomized, controlled trial comparing thoracic paravertebral block to local anesthetic infiltration. Ann Surg Oncol. (2014) 21(3):795–801. 10.1245/s10434-013-3334-6 24165901 PMC3933756

[11] Scibilia G Capobianco SV Bonifacino A Santilli V Paolucci T. Breast cancer rehabilitation: a critical review of clinical practice guidelines and evidence-based medicine in literature. J Rehabil Therapy. (2019) 1(1):11–20.

[12] Santacaterina F Campagnola B Bressi F Zollo L Sterzi S Bravi M. Telerehabilitation in postoperative breast cancer care: systematic review and meta-analysis. JMIR Rehabil Assist Technol. (2025) 12(1):e77161. 10.2196/77161 41364915 PMC12728404

[13] Xue T Zhang L Zhang D. Exercise-based interventions for postoperative rehabilitation in breast cancer patients, a systematic review and meta-analysis of randomized controlled trials. Medicine (Baltimore). (2025) 104(34):e43705. 10.1097/MD.0000000000043705 40859515 PMC12384885

[14] Jacobs A Lemoine A Joshi GP Van de Velde M Bonnet F, PROSPECT Working Group Collaborators. PROSPECT guideline for oncological breast surgery: a systematic review and procedure-specific postoperative pain management recommendations. Anaesthesia. (2020) 75(5):664–73. 10.1111/anae.14964 31984479 PMC7187257

[15] Hussain N Brull R McCartney CJ Wong P Kumar N Essandoh M. Pectoralis-II myofascial block and analgesia in breast cancer surgery: a systematic review and meta-analysis. Anesthesiology. (2019) 131(3):630–48. 10.1097/ALN.0000000000002822 31408448

[16] An R Wang D Liang X-L Chen Q Pang Q-Y Liu HL. The postoperative analgesic efficacy of different regional anesthesia techniques in breast cancer surgery: a network meta-analysis. Front Oncol. (2023) 13:1083000. 10.3389/fonc.2023.1083000 37056343 PMC10088371

[17] Singh NP Makkar JK Kuberan A Guffey R Uppal V. Efficacy of regional anesthesia techniques for postoperative analgesia in patients undergoing major oncologic breast surgeries: a systematic review and network meta-analysis of randomized controlled trials. Can J Anesth. (2022) 69(4):527–49. 10.1007/s12630-021-02183-z 35102494

[18] McNicol ED Ferguson MC. Patient controlled opioid analgesia versus non-patient controlled opioid analgesia for postoperative pain. Cochrane Database Syst Rev. (2015) 2015(6):CD003348. 10.1002/14651858.CD003348.pub3 26035341 PMC7387354

[19] Hudcova J McNicol ED Quah CS Lau J Carr DB. Patient controlled opioid analgesia versus conventional opioid analgesia for postoperative pain. Cochrane Database Syst Rev. (2006) 2006(4):CD003348. 10.1002/14651858.CD003348.pub2 17054167

[20] Buvanendran A Kroin JS. Multimodal analgesia for controlling acute postoperative pain. Curr Opin Anesthesiol. (2009) 22(5):588–93. 10.1097/ACO.0b013e328330373a 19606021

[21] Park JH Lee DW Kim EJ Cho JS. Analgesic efficacy of Acetaminophen/ibuprofen as an adjuvant in patient-controlled analgesia for postoperative pain after oncoplastic breast surgery: a randomized controlled trial. J Clin Med. (2025) 14(22):7901. 10.3390/jcm14227901 41302937 PMC12653202

[22] Forget P Sitter TM Hollick RJ Dixon D Van Maanen A Dekleermaker A. Characterization of preoperative, postsurgical, acute and chronic pain in high risk breast cancer patients. J Clin Med. (2020) 9(12):3831. 10.3390/jcm9123831 33256031 PMC7759784

[23] Zocca J Valimahomed A Yu J Gulati A. A review of recent advances in the management of breast cancer related pain. Breast Cancer Manag. (2018) 7(2):BMT08. 10.2217/bmt-2018-0003

[24] Usul O Dizer B. Pressure injuries related to the positioning of surgical patients in the operating room and identification of associated risk factors: a cross-sectional study. Int Wound J. (2025) 22(7):e70685. 10.1111/iwj.70685 40569268 PMC12199711

[25] Matsen FA III Wyss CR Krugmire RB Simmons CW King RV. The effects of limb elevation and dependency on local arteriovenous gradients in normal human limbs with particular reference to limbs with increased tissue pressure. Clin Orthop Relat Res. (1980) 150:187–95. 10.1097/00003086-198007000-00031 7428219

[26] Dong Y Wang M Li W Zhao K Cui X Yang Y. Effect of dexmedetomidine infusion on postoperative sleep disturbances in women with breast cancer: a monocentric randomized-controlled double-blind trial. Anaesth Crit Care Pain Med. (2024) 43(3):101358. 10.1016/j.accpm.2024.101358 38365169

[27] Yang B Li J Feng D Gong J Yang Y Cai X. Latent profiles and determinants of postoperative sleep quality in elective surgery patients. Sci Rep. (2025) 15(1):617. 10.1038/s41598-024-84896-x 39753664 PMC11698858

[28] Finan PH Goodin BR Smith MT. The association of sleep and pain: an update and a path forward. J Pain. (2013) 14(12):1539–52. 10.1016/j.jpain.2013.08.007 24290442 PMC4046588

[29] Haack M Simpson N Sethna N Kaur S Mullington J. Sleep deficiency and chronic pain: potential underlying mechanisms and clinical implications. Neuropsychopharmacology. (2020) 45(1):205–16. 10.1038/s41386-019-0439-z 31207606 PMC6879497

[30] Niyonkuru E Iqbal MA Zhang X Ma P. Complementary approaches to postoperative pain management: a review of non-pharmacological interventions. Pain Ther. (2025) 14(1):121–44. 10.1007/s40122-024-00688-1 39681763 PMC11751213

[31] Gadiraju GK Prospero MR Tobias F Malek AJ Reiche E Broyles JM. Understanding the impacts of surgical drains on postoperative pain and quality of life. Plast Reconstr Surg Glob Open. (2025) 13(1):e6474. 10.1097/GOX.0000000000006474 39872083 PMC11771670

[32] Malchrowicz-Mośko E Nowaczyk P Wasiewicz J Urbaniak T Siejak W Rozmiarek M. The level of kinesiophobia in breast cancer women undergoing surgical treatment. Front Oncol. (2023) 13:1010315. 10.3389/fonc.2023.1010315 PMC9932589 36816937

[33] Kim S-Y Shin Y-S. A comparative study of 2-hour interface pressure in different angles of laterally inclined, supine, and fowler's Position. Int J Environ Res Public Health. (2021) 18(19):9992. 10.3390/ijerph18199992 34639293 PMC8507824

[34] Asiri S. Turning and repositioning frequency to prevent hospital-acquired pressure injuries among adult patients: systematic review. Inquiry. (2023) 60:00469580231215209. 10.1177/00469580231215209 38050921 PMC10699153

[35] Azizoddin DR Soens MA Beck MR Flowers KM Edwards RR Schreiber KL. Perioperative sleep disturbance following mastectomy: a longitudinal investigation of the relationship to pain, opioid use, treatment, and psychosocial symptoms. Clin J Pain. (2023) 39(2):76–84. 10.1097/AJP.0000000000001090 36650603 PMC9968504

[36] Rampes S Ma K Divecha YA Alam A Ma D. Postoperative sleep disorders and their potential impacts on surgical outcomes. J Biomed Res. (2020) 34(4):271. 10.7555/JBR.33.20190054 PMC7386412 32519977

[37] Gillespie BM Walker RM Latimer SL Thalib L Whitty JA McInnes E. Repositioning for pressure injury prevention in adults. Cochrane Database Syst Rev. (2020) 2020(6):CD009958. 10.1002/14651858.CD009958.pub3 PMC7265629 32484259

[38] Perrenoud B Maravic P Delpy P. Pressure injury prevention in the operating unit of a Swiss university hospital: a best practice implementation project. JBI Evid Implement. (2023) 21(1):46–57. 10.1097/XEB.0000000000000341 36374987 PMC10010690

[39] Mak AF Zhang M Tam EW. Biomechanics of pressure ulcer in body tissues interacting with external forces during locomotion. Annu Rev Biomed Eng. (2010) 12(1):29–53. 10.1146/annurev-bioeng-070909-105223 20415590

